# Friends or Foes? Relational Dissonance and Adolescent Psychological Wellbeing

**DOI:** 10.1371/journal.pone.0083388

**Published:** 2014-02-03

**Authors:** Lyndal Bond, Dean Lusher, Ian Williams, Helen Butler

**Affiliations:** 1 Centre of Excellence in Intervention and Prevention Science, Melbourne, Victoria, Australia; 2 Swinburne Business School, Swinburne University of Technology, Melbourne, Victoria, Australia; 3 Centre for Adolescent Health; Murdoch Children’s Research Institute, Melbourne, Victoria, Australia; 4 School of Education, Australian Catholic University Limited, Australia, Melbourne, Victoria, Australia; Cinvestav-Merida, Mexico

## Abstract

The interaction of positive and negative relationships (i.e. I like you, but you dislike me – referred to as *relational dissonance*) is an underexplored phenomenon. Further, it is often only poor (or negative) mental health that is examined in relation to social networks, with little regard for positive psychological wellbeing. Finally, these issues are compounded by methodological constraints. This study explores a new concept of relational dissonance alongside mutual antipathies and friendships and their association with mental health using multivariate exponential random graph models with an Australian sample of secondary school students. Results show male students with relationally dissonant ties have lower positive mental health measures. Girls with relationally dissonant ties have lower depressed mood, but those girls being targeted by negative ties are more likely to have depressed mood. These findings have implications for the development of interventions focused on promoting adolescent wellbeing and consideration of the appropriate measurement of wellbeing and mental illness.

## Introduction

It is widely accepted that social connectedness is associated with physical and mental health [1]–[5]. Studies have shown, for example, that social connectedness promotes positive mental health through increased access to social support and an enhanced sense of coherence and purpose in life [6]. For young people, connectedness to family and school emerge as key protective factors associated with lower rates of engagement in health risk behaviours and with better mental health and educational outcomes [7]–[13]. While the positive influence of social networks on health is often cited, social relationships can also have negative impacts [14]–[16]. It is clear that strained social ties, such as those that may be experienced in marital relationships, parent-child relationships, or peer relationships, can undermine both physical and mental health.

While studies have examined separately the positive and negative influences of social relationships on health, it is unclear how these different aspects of social ties might work *together* [6]. We have found no studies that examine both types of relationships simultaneously with regard to their effects on mental health and wellbeing. Further, methodologically most studies have failed to account for the endogenous, self-organizing processes inherent in human social networks, a consequence of which may be to overestimate associations between individual attributes and network structures. In the present paper we seek to address the limitations of previous research noted above by examining both positive and negative social relationships together, along with their association with mental health and wellbeing, and by using a particular class of statistical model for social networks, which takes account of network structure characteristics.

### Positive and Negative Effects of Peer Networks

Adolescents typically spend increasingly more time with their peers than with their families as they make the transition from childhood to adulthood. Friends can therefore become important sources of social influence, both positive and negative [14], [17]–[20]. Whether an individual is isolated or enmeshed, their wider peer network(s) may determine the degree to which they are exposed to a broad range of behaviours, not all of which will be health promoting [21]. Accordingly, research examining the effects of young people’s social connectedness to peers should examine both the likely positive and negative effects on young people’s health and wellbeing. For example, studies examining youth substance use and other health risk and anti-social behaviours have reported positive effects of affiliations with pro-social peers [15], [22] and detrimental effects of negative peer influences [20], [23]. Yugo and Davidson [24] found high levels of peer connectedness were related to good health and self–worth, but were also related to alcohol, tobacco and marijuana use.

Studies examining associations between social relationships and adolescent mental health present a similarly complex picture. Positive friendship qualities (such as intimacy and support) have been found to be inversely related to anxiety but not to depressed mood, while negative friendship qualities (such as conflict, pressure and exclusion) appear to be associated with increased levels of both anxiety and depression [25]. In a study of Israeli-born adolescents, Walsh, Harel-Fisch, and Fogel-Grinvald [26] reported that peer support was protective for internalising behaviour problems where parental support was also high, but was a risk factor for adolescents with low parental support. They also found that while having friends was positively related to mental health, spending too much time with others was related to poor mental health outcomes.

### Limitations of Previous Research

Three major limitations from previous research can be identified. First, many studies fail to differentiate between positive and negative aspects of mental health. While it is claimed that social relationships are associated with good mental *health*, most studies test this assertion using measures of mental *illness* [26]–[28]. As stated by the World Health Organisation, however, mental health is not just the absence of problems or illness, but is “a state of wellbeing in which every individual realises his or her own potential, can cope with the normal stresses of life, can work productively and fruitfully, and is able to make a contribution to her or his community” [29]. The present study adopts this fuller definition of mental health by including measures both of psychological wellbeing and of mental ill health.

A second limitation of previous studies is their typical focus on a single type of social tie. As previously indicated, earlier research has tended to examine positive relations only, or negative relations only, but not the two together. It is reasonable to assume that one social network does not operate in a vacuum from another [30], Studies in organisational settings have noted how one type of relation may be dependent upon another [31], finding that, for instance, A seeks advice from B, B socializes with A outside of work (e.g. see [32]), or “A trusts B, B finds A difficult to work with [33]. Interdependencies between ties and interaction between positive and negative relationships may therefore be important for a thorough examination of associations between social networks and mental health. The present study examines the influence of both positive and negative social ties on measures of mental health.

A final shortcoming of past research relates to limitations in the statistical methods typically used to examine social relationships. Many commonly used methods fail to take account of dependencies between social ties. Individuals within a social network are not unrelated “units of analysis”, but instead are interdependent entities engaged in social relations [34]. One of the ways in which complexity and dependencies are ignored is by disregarding network self-organisation. *Network self-organisation* refers to the creation of ties and network structures that arise due to the presence of other network ties. Examples include the notion of “you scratch my back, I’ll scratch yours” (the general principle of reciprocity [35]) and “a friend of a friend is a friend” (the principle of triadic or path closure). Such characteristics are not considered attributes of individuals, but instead are thought of as structural network effects. In all such instances, the presence of a relationship is *dependent* upon the presence of other relations (e.g. it is only *because* you have scratched my back that I will scratch yours). Statistical models, such as linear regression, that treat social ties as attributes of individuals cannot account for these important inter-dependencies – indeed, many such methods assume *independence* of observations – yet controlling for such dependency effects is considered critical in any analysis of social networks [36]–[38]. These methods therefore disregard network self-organisation and may inadvertently overestimate the importance of individual attributes and make inaccurate conclusions about the effects of social ties [39]. In the present paper we used exponential random graph models (ERGM) as a preferred method to take account of complex dependencies in the data and avoid over-estimating the effects of individual attributes with regard to network tie formation.

### Association of Social Networks to Mental Health and Wellbeing

Three specific types of association between the structure of young peoples’ social networks and their mental health and wellbeing can be identified in the literature. We next review these associations and propose empirical hypotheses to examine in the present paper.

#### Number of network ties

Associations between network size and mental health are complex. On one hand, direct associations between social network size and mental health have been reported in adult populations, such that a large number of social ties appears related to mental health benefits [27]. Conversely, it has been argued that having too many friends may lead to strain and stress in efforts to maintain multiple relationships [5], [21], [27]. It is possible that the influence of network size may be offset by network density: small, dense networks might provide adequate social support, while large networks might be less integrated and therefore offer less support [5], [21], [27].

Haas and colleagues [21] examined the relationship between network structures and general health amongst adolescents. They proposed that individuals with health problems may have smaller social networks due to: i) fewer opportunities to form and maintain friendships; ii) peers being less willing to engage with them due to the possible stigma of associating with a sick friend; and iii) lack of reciprocity of social support. The authors found that young people who reported poor health did in fact have fewer network ties and weaker friendships.

In a study of friendship networks and adolescent depression, Ueno [27] examined the effects of a range of social integration measures (including total number of friends, egocentric density, friendship reciprocity and position in school-wide networks) on depressive symptoms. Ueno proposed that small networks might not provide enough social support, leading to feelings of isolation and reduced social worth. He found that network size was the strongest predictor of depressive symptoms, such that those with few friendship ties were more likely to experience depression.

In a follow-up study, Falci and McNeely [5] examined the association between adolescent depressive symptoms and two dimensions of network structure: social integration (measured by number of ties, type of tie, and frequency of contact) and network cohesion (density of network ties). Results showed a U-shaped relationship between number of friends and depressive symptoms: those with very small and very large networks reported more depressive symptoms. The study also found several gender differences. For girls, those in large and fragmented networks reported the highest levels of depressive symptoms, while those in cohesive networks showed no such association with depression. Conversely, boys in large, fragmented networks showed lower levels of depressive symptoms. Cohesive networks whether small or large were associated with depressive symptoms.

While the literature paints a complex picture of the ways in which social relationships may relate to mental health, it is apparent that friendships (positive social relationships) may be associated with positive mental health. In the present study we therefore hypothesise that, relative to others in the same network *young people who receive high numbers of friendship nominations will have higher psychological wellbeing and/or lower depressed mood* (hypothesis 1, *H1*).

#### Peer rejection and negative relationships (antipathies)

A second way that young people’s social networks might affect their mental health and wellbeing may be through negative relationships, or peer rejection. Peer rejection can take many forms, such as deliberate exclusion, ignoring, teasing and bullying, and can include both the absence of positive social ties and the presence of negative social ties. A more specific definition of peer rejection describes “being unilaterally disliked and being involved in mutually antipathetic relationships” [40].

Peer rejection in the form of social isolation has been shown to be related to depressive symptoms [5] and to substance use [16], though peer rejection in these studies has been defined as the absence of positive social ties rather than the presence of negative social ties. Others have distinguished peer rejection as “being unilaterally disliked and being involved in mutually antipathetic relationships” [40], and therefore taken a more specific analysis of negative social ties. Card and Hodges found that mutual antipathies may be a pre-cursor to feelings of victimization [40]. Adolescents who have mutual antipathies are also more likely to be withdrawn or engage in antisocial behaviour [41]. Finally, negative social interactions both with friends and with family appear to be associated with depression [42]. The balance of literature on the topic clearly suggests that the association between adolescent depression and social network structures is important [43], with the general finding that for negative social ties “less is better”, or more precisely, “more is worse”. We therefore hypothesise in the current study that, relative to others in the same network *young people who receive high numbers of dislike nominations will have lower psychological wellbeing and/or higher depressed mood* (hypothesis 2, *H2*).

#### Relational dissonance: The dyadic exchange of a positive and a negative social tie

In this paper we extend conceptualisations of peer rejection relationships, and include a new term – relational dissonance, which may be another way in which social networks are related to depression and/or reduced wellbeing. Relational dissonance, or liking others but being disliked by them in return (as opposed to just not being liked in return) exemplifies a case in which a friendship tie is given from A to B, but reciprocated (or perhaps more accurately, exchanged) with a dislike tie from B to A. Relational dissonance is the interaction between two different types of social network ties (i.e., liking and disliking) that produces the relationship of interest, not just one network or the other, thereby making this a multivariate network effect. The term relational dissonance can be seen as derivative to some degree from cognitive dissonance of Heider’s [44] Balance Theory, a psychological state of stress that can result from imbalanced social relations. We are not just measuring whether you consider someone a friend and they do not reciprocate. Instead, we are measuring the discordance between one person considering another a friend, and the person actually disliking the person who offers friendship (i.e. exchanges a friendship tie with a negative tie).

Research that supports our idea of relational dissonance is limited. We know that friendship instability is associated with increased depressed mood [45]. Further, we know that the relationship between friendship and aggressive attitudes has been studied [46], but not specifically the multivariate interaction between friendship and negative social ties. The only study of which we are aware that has touched upon dissonant social ties is by Zhao and Robins [33] who noted in an organisational setting the dyadic exchange of network ties of advice and work difficulty (i.e., A trusts B, B finds A difficult to work with). We have found no studies that have examined such dissonant relationships and mental health. In this case, our final hypothesis is that relational dissonance (i.e. A likes B, B dislikes A) may be stressful for the young people involved. We propose that, relative to others in the network that *young people with relationally dissonant relations (i.e. A likes B, B dislikes A) will be lower in wellbeing and/or higher in depressed mood (hypothesis 3, H3).* We assess these hypotheses by examining young people’s positive and negative social networks and their associations with positive and negative mental health, simultaneously using multivariate exponential random graph models.

As previous studies (e.g. [5]) have reported gender differences between social networks and depressive symptoms, we have tested these hypotheses for males and females separately.

## Methods

### Setting

The context in which we explored the association between social networks and mental health was an independent, co-educational secondary school in the outer suburbs of metropolitan Melbourne, Australia. This school was selected because of its involvement in a previous research project and its request for further research on school climate. All students in Year 8 (13–14 year olds) were invited to participate in the study. Most secondary schools in this part of Australia span from Year 7 (12–13 years of age) to Year 12 (17–18 years of age). In total the school had approximately 900 students. From a pool of 165 Year 8 students, 130 (79%) were granted parental consent and took part in the study. Complete data for the present analyses was obtained for a total of 120 students (73% of the original sample). Females comprised 51% of the sample. Sixty-three percent had one or both parents with a tertiary qualification and 5% spoke a language other than English (LOTE) at home.

### Procedure

Ethics approval was obtained from the Royal Children’s Hospital Human Research Ethics Committee. Written consent from parents was required for students to participate in the survey and in line with standard ethics procedures, we made it clear to parents that their child’s participation was voluntary. Students were also asked for their consent on the day of the survey. Again we made it clear that their participation was voluntary. Consenting students self-completed a 40-minute web-based survey during class time.

### Measures/Data

#### Psychological wellbeing

The 14-item Psychological Wellbeing (PWB) subscale of the Mental Health Inventory was used to assess psychological wellbeing. The Inventory has been confirmed as applicable to adolescent samples [47], [48]. The PWB subscale includes items relating to general positive affect (e.g. How much of the time, during the past month, have you felt cheerful, light-hearted?) and emotional ties (e.g., During the past month, how much of the time have you felt loved and wanted?). For each item, respondents are asked to consider the extent to which they have felt this way during the previous month. Items are rated using a six-point Likert scale with varying response sets; responses are summed across the 14 items such that higher PWB scores equate to greater wellbeing (possible range 14 to 84). Psychometric properties of the PWB subscale are acceptable, with internal consistency (Cronbach alpha) of 0.92 and 10-week test-retest reliability of 0.69 [48]. Internal consistency (Cronbach alpha) in the present study was found to be 0.93.

#### Depressive symptoms

Depressive symptoms were measured using the short form of Angold and Costello’s Mood and Feelings Questionnaire (SMFQ), a self-report scale designed to identify clinically meaningful symptoms of depression amongst child and adolescent populations [49]. Comprising a subset of 13 items from the original 33 (long form), the SMFQ is a widely used, uni-dimensional scale that taps DSM diagnostic criteria for major depressive disorder. Items include somatic (e.g. I am restless, find it hard to sit still), affective (e.g. I feel miserable or unhappy), cognitive (e.g. I think I can never be as good as others) and behavioural (e.g. I cry easily) aspects of depression. Respondents are asked to indicate using a three-point Likert scale (0 = rarely or never, 1 = sometimes, 2 = very often) how frequently they have experienced the symptom described during the previous two-week period. Items are summed to provide a total score, with high scores indicating a greater number of symptoms. Internal reliability of the SMFQ has been reported as 0.87 [50] and criterion validity has been established with both the Children’s Depression Inventory and the Diagnostic Interview Schedule for Children depression scales [49]. Internal consistency (Cronbach alpha) in the present study was found to be 0.99.

#### Socio-economic status (SES)

Parental education was treated as an indicator of socio-economic status (SES). Students were asked whether their parents undertook tertiary education (1) or not (0). Students were also asked whether they spoke a language other than English (LOTE) at home. Due to the very small numbers for LOTE we did not include this variable in further analyses.

#### Social network questions

To elicit social relations amongst participants, students used the standard social network roster method [51], which contained a complete list of all Year 8 students. Students completed the survey using student numbers, and not names, so that the data was de-identified. For positive social relations (hereafter the friendship network) participants were asked to nominate:

1. Who is in your close group of friends?

In this binary and directed friendship network, a tie *x_ij_* = 1, otherwise = 0.

A second social network (hereafter the *disliking* network) was constructed by combining responses to two questions capturing information about negative relationships.

2a. Who do you not get along with?
2b. Which students would you choose not to have lunch with?

In this binary and directed disliking network, a nomination in either question 2a and/or 2b led to a tie *x_ij_* = 1, otherwise = 0.

So in summary, we have two binary networks – one for friendship and one for disliking, which we analyse simultaneously. The network is directed, such that A choosing B is separate from B choosing A, and a 1 indicates the presence of a directed friendship/dislike tie, and a 0 the absence of a directed friendship/dislike tie.

### Analysis

#### Exponential random graph models (ERGM)

The network analyses employ exponential random graph models (ERGM). Originally proposed by Frank and Strauss [52] and later developed by others [53]–[58], these are a particular class of statistical model for social networks. ERGMs are statistical models for network structure [59], [60]. In an ERGM, network substructures are represented by parameters that reflect positive or negative tendencies for these network substructures to be present in the observed network (i.e., the network data we collected). Different substructures (or network configurations) represent different theoretical assertions regarding why network ties are formed, such as via reciprocity, preferential attachment, or some individual quality of the network actors. ERGMs differ from linear regression models because ERGMs do not assume independence of observations, but instead use specific dependence assumptions to take into account that the presence of one network tie can affect the presence or absence of another. This aligns method more closely with theory, for the social is all about interdependence of relations, which makes ERGM a preferred method for the statistical analysis of network data [60]. For a general introduction to ERGM see Robins et al. [59], and for a comprehensive review see Lusher et al [60]. For a detailed discussion of multivariate ERGM, see Wang [61].

ERGM is a tie-based mode for the prediction of network ties, not a social influence-type model for the prediction of actor attributes [55]. ERGM is most often used for cross-sectional data thus causal claims cannot be made. Nonetheless, ERGM functions as a pattern recognition algorithm, identifying regularities in social network structures, and associations between social network ties and individual attributes [56].

Specifically, we employed *multivariate* ERGM as we wish to examine two cross-sectional networks – friendship and dislike – simultaneously. ERGM measures the complexities of nested social structures as well interdependencies in the data [62] using Markov Chain Monte Carlo Maximum Likelihood Estimation (MCMCMLE) [57]. We note that very few studies have used multivariate ERGM [31]–[33], [63].

#### Empirical model specifications

**Figure 1:**
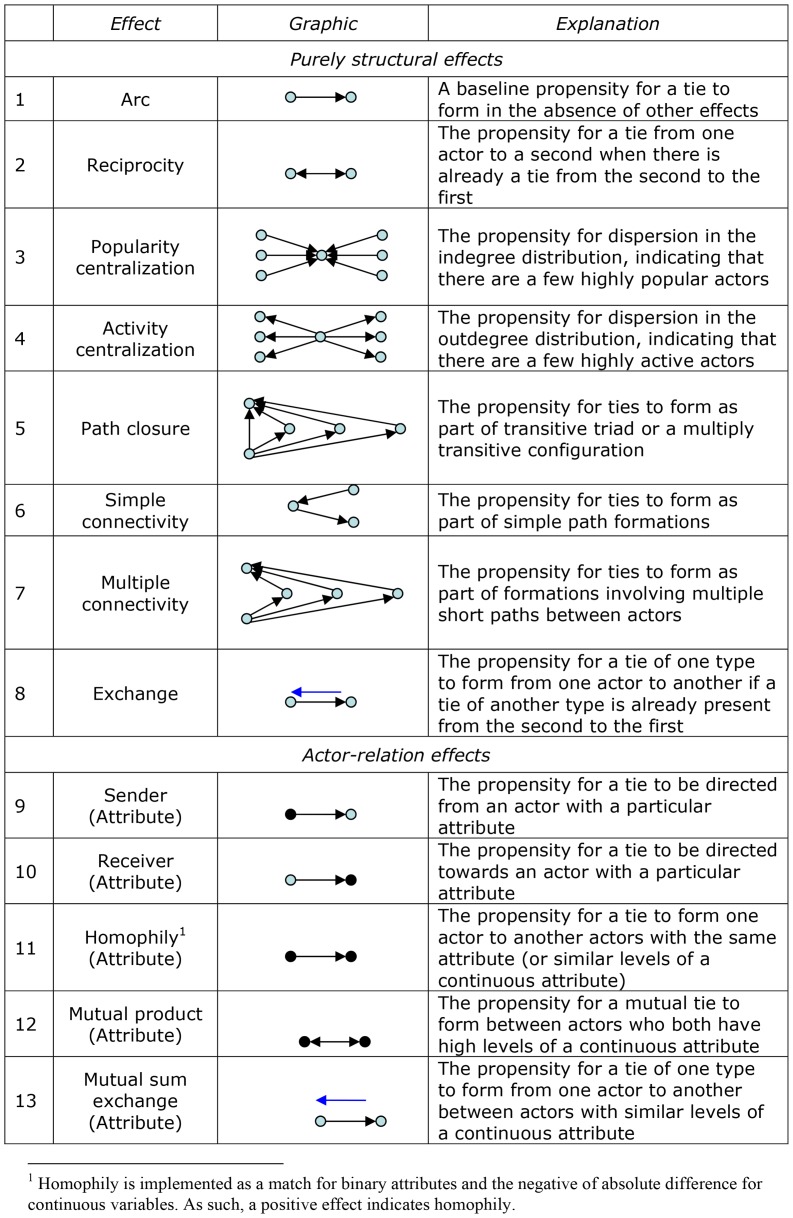
Parameters used in multivariate exponential random graph models.

The network parameters included in our models, including a graphical representation and explanation, are presented in Figure 1. Each parameter is essentially an hypothesis about how social ties are formed. In some ways, we might think of the dependent variable of an ERGM as the presence of a network tie. An ERGM is a model “…for a class of mutually interdependent variables that may also depend on another class of exogenous variables” (60: p. 106). For this reason it is crucial to “specify the dependence assumptions appropriately because the proposed dependencies determine the form of the configurations parameterized in the model” (60: p. 106).

#### Variables and measures

The two principal types of effect in ERGM are actor-relation effects and purely structural effects. Actor-relation effects account for network ties that arise due to actor attributes, which can occur in various ways. A sender effect indicates whether individuals with particular attributes are more likely to send social ties, and for the friendship network may reflect deference to others, or an effort to embed oneself within a social context – thus making many ties and hoping some “stick” (e.g., come to fruition as reciprocal ties). A receiver effect refers to the propensity of individuals with particular attributes to receive social network nominations, and is clearly indicative of prominence and prestige within the network. A third effect is homophily, or ‘birds of a feather flock together’ [22], which simultaneously examines the attributes of the sender and receiver of a tie and indicates the degree to which individuals choose similar others as network partners, either with asymmetric (effect 11) or mutual ties (mutual product attribute, effect 12). Finally, mutual sum exchange represents the exchange of network ties (friendship and dislike) and it association with actor attributes. In each of these effects, network relations arise because of the attributes of the people in the network.

The attribute variables were implemented into our social network models in the following way. For PWB, SMF and SES we include separate sender, receiver, and homophily effects. Importantly, homophily effects only represent one person choosing another with the same/similar attribute, and not the tendency of pairs to mutually choose one another. To address the latter issue, we also included one mutual product effect for friendship and PWB, and one for dislike and SMF which measure the tendency of people who choose one another to both be high on a certain attribute (in this case, PWB and SMF). Finally, we included one mutual sum exchange effect (i.e. the exchange of friendship for dislike) for PWB, and one for SMF. That is, where A selects B as a friend, but B selects A as someone they dislike we look at the combined (i.e., added) PWB/SMF of the two actors.

*Purely structural effects* account for endogenous processes of network self-organization, which in Table 1 are represented by effects 1 through 8. Such purely structural network effects refer to network structures that do not depend on actor attributes or other exogenous factors, but instead depend upon the presence or absence of other network ties. The notion of reciprocity reflects a purely structural network effect because reciprocation depends upon the presence of the other initial tie (e.g., it is because you have scratched my back that I scratch yours). So network self-organization reflects the fact that the presence of ties can encourage other ties to come into existence [60]. Such effects are included as control variables in our analyses. For the friendship network we included effects 1 through 7, and for the disliking network we included effects 1–4 and 6–7. The multivariate effect, 8, jointly assessed the exchange of friendship and dislike ties.

**Table 1:** Basic statistics for friendship and disliking networks for boys and girls.

	Boys		Girls	
	Friendship	Dislike	Friendship	Dislike
Density	0.07072	0.01870	0.09044	0.02459
Number of ties	242	64	331	90
Mean In\Out degree^1^	4.10169	1.08475	5.42623	1.47541
Minimum outdegree	1	0	1	0
Maximum outdegree	14	11	14	8
Minimum indegree	0	0	0	0
Maximum indegree	10	6	10	10
Number of reciprocated pairs	71	3	115	5
Number of pairs exhibiting exchange of friendship & dislike	7		20	

1 Indegree refers to the number of times an actor is selected by all other network actors, while outdegree refers to the number of nominations an actor makes to all others.

Finally, multivariate network analyses were conducted using the XPNet software [27], [64], simultaneously examining the friendship and dislike networks separately for boys and girls, though the same model parameters were used for each analysis. In terms of statistical power, although we have only in one analysis 59 boys and another 61 girls, the number of data points in these tie-based models is the number of possible social ties, not the number of participants/network actors. Excluding self-nominations, the number of data points is 2n(n−1) or 6,844 for boys and 7,320 for girls. Calculation of the conditional log odds comes from counts of such statistics in the data multiplied by the parameter estimates resulting from the model.

## Results

On average there was no statistically significant difference in psychological wellbeing (PWB) for girls (M = 58.2, SD = 12.6) and boys (M = 61.1, SD = 11.6), *t*(118) = −1.31, p = .19. However, girls scored significantly higher on the measure of depressive symptoms (M = 6.0, SD = 5.1) relative to boys (M = 4.0, SD = 5.0), *t*(118) = 2.10, p<.05). The pattern of friendship network ties tended to cluster according to gender (girl-girl = 50%; boy-boy = 39%; girl-to- boy = 6%; and boys-to-girls = 5%). A similar pattern was found for dislike relations (girl-girl = 49%; boy-boy = 37%; girl-to-boy = 8%; and boy-to-girl = 6%). Given very few cross-gender nominations and because there are gender differences in the prevalence of depressive symptoms, subsequent analyses examined the social networks of boys and girls separately. We begin with some basic descriptive network statistics in Table 1.

As seen from Table 1, the number of relationally dissonant ties (20 for girls, 7 for boys) was greater than the number of mutual antipathy ties for both girls (5) and boys (3), which is first evidence that such relationships might be important. Every student nominated at least one friend, but not every student was nominated by another as being a friend. We also note that the average degree was almost four times higher for friendship than dislike for both genders.

The parameter estimates and associated standard errors from the multivariate ERGM analyses are presented in Table 2. For the multivariate ERGMs all estimates showed good convergence of the MCMCMLE estimation algorithm with convergence t-statistics <0.1 and the goodness of fit (GOF) statistics were excellent for all observed network effects (i.e. t-statistics <0.1). Estimates > = 2 SEs are regarded as statistically significant, indicating that a network effect occurs greater than chance levels, given the other effects in the model.

**Table 2:** Network effect estimates (with SE) for separate (gender) multivariate ERGM analyses (friendship and disliking) and psychological wellbeing (PWB) and depressed mood (SMF).

				Boys	Girls
Network parameter				Estimate (SE)	Estimate (SE)
**Hypothesized effects**					
*Actor-relation effects*					
[H1]		Receiver friendship (PWB)		0.007 (0.010)	−0.001 (0.010)
		Receiver friendship (SMF)		−0.026 (0.026)	0.001 (0.022)
[H2]		Receiver dislike (PWB)		0.004 (0.014)	0.001 (0.010)
		Receiver dislike (SMF)		0.050 (0.040)	0.085 (0.032)*
[H3]		Mutual sum exchange (PWB)		−0.058 (0.026)*	−0.011 (0.020)
		Mutual sum exchange (SMF)		−0.121(0.094)	−0.105 (0.049)*
**Control effects**					
*Actor-relation effects*					
Friendship network					
		Sender (PWB)		−0.006 (0.009)	0.012 (0.010)
		Sender (SMF)		−0.020 (0.024)	0.006 (0.020)
		Sender (SES)		−0.170 (0.164)	0.022 (0.158)
		Receiver (SES)		−0.088 (0.165)	0.033 (0.167)
		Homophily (PWB)		−0.001 (0.007)	0.001 (0.006)
		Homophily (SMF)		0.002 (0.022)	0.017 (0.012)
		Homophily (SES)		0.313 (0.168)	0.071 (0.212)
		Mutual product (PWB)		0.0002 (0.0002)	−0.0001 (0.0002)
Disliking network					
	Sender (PWB)			0.023 (0.012)	−0.009 (0.011)
	Sender (SMF)			0.023 (0.036)	−0.039 (0.034)
	Sender (SES)			−0.449 (0.298)	0.220 (0.254)
	Receiver (SES)			−0.710 (0.337)*	−0.122 (0.267)
	Homophily (PWB)			−0.005 (0.016)	−0.009 (0.010)
	Homophily (SMF)			0.043 (0.044)	0.032 (0.033)
	Homophily (SES)			0.718 (0.599)	0.064 (0.453)
	Mutual product disliking (SMF)			−0.116 (0.138)	0.006 (0.004)
*Purely structural effects*					
Friendship					
	Density			0.861 (1.453)	−0.967 (1.408)
	Mutual			0.488 (0.803)	2.049 (0.746)*
	Simple connectivity			−0.092 (0.048)	−0.051 (0.033)*
	Popularity centralization			−0.945 (0.332)*	−1.104 (0.459)*
	Activity centralization			−0.668 (0.289)*	−0.627 (0.322)
	Path closure			1.362 (0.125)*	1.485 (0.146)*
	Multiple connectivity			−0.371 (0.072)*	−0.161 (0.043)*
Disliking network					
	Density			−6.715 (1.354)*	−5.743 (1.156)*
	Mutual			2.862 (0.804)*	0.895 (0.724)
	Popularity centralization			0.617 (0.244)*	0.926 (0.209)*
	Activity centralization			0.872 (0.234)*	0.692 (0.202)*
	Multiple connectivity			−0.205 (0.107)	0.015 (0.048)
Friendship and Disliking networkmultivariate effects					
			Exchange	8.392 (3.382) *	3.702 (2.701)

In Table 2 we have placed effects that relate to our hypotheses at the top of the table, with control network effects below.

H1 proposed that students receiving many friendship ties would score highly on PWB and/or score low on depressed mood. In such a case the actor-relation receiver effect for friendship ties with PWB should be positive and significant (indicating that students who receive many friendship ties should be high on PWB), and/or the receiver effect for depressed mood should be negative and significant (indicating that students who receive many ties have low scores on depressed mood). We found no such significant receiver effects for psychological wellbeing or depressive symptoms for either boys or girls.

For H2 we predicted that students receiving many dislike ties would be low in PWB (i.e. a negative and significant sender effect) and/or high in SMF (i.e. a positive and significant sender effect). We found no support for the boys for this hypothesis. However, there was support for girls for dislike ties with a positive and significant receiver effect for depressed mood (SMF). This indicates that for girls being highly unpopular (i.e. disliked by many others) is associated with increased depressed mood.

Finally, H3 related to relational dissonance – that is, a discrepant dyadic relationship in which student A nominates B as a friend, and student B nominates A with a dislike relation. Separate multivariate mutual sum exchange effects were included for PWB and SMF. For boys, there was significant and negative parameter for PWB, which indicates that relationally dissonant ties are likely to occur for boys when the sum of the boys’ PWB scores is low. For girls, there was a significant and negative mutual sum exchange effect for discrepant relationships and depressed mood. The interpretation is that the exchange of friendship and disliking relations is likely to occur for small values of depressed mood.

To look at this effect from another angle we can demonstrate how possible high and low values of PWB are differentially associated with the presence of a relationally dissonant tie between a pair of actors in the network. More formally, we examine the *conditional log-odds* relating to attributes of observing the exchange of a friendship and dislike tie for scores on the PWB variable. To do this, we multiply the parameter estimate values from Table 2 with possible values of the psychological wellbeing variable for selected pairs of actors to see how probable a tie is between students. The values chosen represent the highest and lowest non-zero observed scores. The results of possible combinations are presented in Table 3.

**Table 3:** The conditional log-odds of observing an exchange of friendship and dislike ties (i.e., relational dissonance) between boys for various values of the attribute PWB (the values chosen represent the lowest and highest observed scores).

Friendship network			Dislike network		
Possible Tie Configuration	Sender (PWB)	Receiver (PWB)	Sender (PWB)	Receiver (PWB)	Conditional Log-odds
**A**	20	20	20	20	−1.8
**B**	20	79	79	20	−3.1
**C**	79	79	79	79	−7.0
**D**	79	20	20	79	−4.9

The conditional log-odds from Table 3 are highest in configuration A when PWB is low (i.e. 20) for both boys in the dyad, and least likely for configuration C when PWB is high for both boys. As such, boys with relationally dissonant ties are both likely to be low on PWB.

Table 4 presents the conditional log-odds of observing the exchange of a friendship and dislike tie between girls for scores on the SMF variable. What we see here is that the most likely values of depressed moods for both girls in such dyads are low.

**Table 4:** The conditional log-odds of observing an exchange of friendship and dislike ties between girls (i.e., relational dissonance) for various values of the attribute SMF (the values chosen represent the highest and lowest non-zero observed scores).

Friendship network			Dislike network		
Possible Tie Configuration	Sender (SMF)	Receiver (SMF)	Sender (SMF)	Receiver (SMF)	Conditional Log-odds
**A**	1	1	1	1	−0.2
**B**	1	22	22	1	−4.2
**C**	22	22	22	22	−3.5
**D**	22	1	1	22	−1.5

### Control Effects

For boys, there was a non-significant but positive trend for those who make many friendship nominations to have high PWB. However, we found no significant effect of homophily of mental health: neither boys nor girls were more likely to choose as friends others of similar wellbeing or depressed mood to themselves. We found that a significant and negative receiver effect for SES, indicating that boys with low SES were selected more with dislike ties than high SES boys.

Finally, there were many significant endogenous network effects, indicating that network self-organization is an important explanation for social tie formation, above and beyond any attribute effects such as mental health or SES, and therefore must be taken into consideration. For both friendship networks we see significant path closure effects, indicating that students congregate in triadic clusters indicative of small groups. Importantly, we note that there were significant and positive popularity centralization and activity centralization effects for both genders. This means that there are some students who are highly unpopular (i.e. nominated by many as disliked) and also some who dislike many other students (i.e. nominate many others as students they dislike), but that such effects are above and beyond such effects of the attributes we have included in this model.

## Discussion

This paper examined the associations between relational dissonance experienced by young people and their mental health. We are unaware of any published studies examining these associations between such discrepant relationships and wellbeing or depression. In direct contrast to other studies (e.g. [27]) we found no association between increasing friendships nominations and mental health. The ability of ERGM to accommodate network self-organization is quite likely the reason for this null finding, because such an analysis does not over-estimate the importance of individual-level variables with regards to network ties, highlighting the need for appropriate analytic methods for social network data with complex dependencies.

Further, we found that receiving many negative ties was associated with depressed mood for girls but not boys. This finding is congruent with other studies which have reported associations between negative aspects of friendships and anxiety and depressed mood [25], [65] and may contribute to greater prevalence of depressive symptoms for girls in this study and others.

Importantly, beyond our first two hypotheses, and controlling for a range of other explanations for the presence of network ties, we have found evidence for boys and girls, although in different directions, for our introduced concept of relational dissonance and its association with mental wellbeing. This provides evidence that such discrepant relationships may be very important to adolescent mental health, and highlights the value of examining multiple networks simultaneously. The current result goes beyond the lack of reciprocation of friendship ties, or the receipt of dislike ties.

For boys, while we found no support for the first two hypotheses, relational dissonance was associated with low psychological wellbeing for both dyad members. The implication of this finding is that in a dyad in which boys do not view the relationship evenly, both boys are likely to have low positive psychological wellbeing. However, it does not mean that both boys are depressed, as we included a separate effect for this precise possibility and found it was non-significant. It is possible that this is a selection effect, where a boy chooses another of similarly low psychological wellbeing to be a friend, but is rejected by the other who may find such a friendship unattractive because they would prefer to interact with happier others. Of course, it may be there is also an influence effect, such that the being liked by someone with low psychological wellbeing brings one’s own mental health down. We do note that it was not the presence of negative mental health, but the absence of positive psychological wellbeing for relational dissonance, and this highlights the importance of measuring these two distinct constructs.

In contrast, for girls we found that positive psychological health was not associated with friendship or dislike ties, but that depressed mood was associated with being highly disliked (i.e. receiving many dislike nominations). Further, girls in relationally dissonant ties were both likely to be low in depressed mood. For the girls, these two effects must be read in conjunction, one given the other. As such, highly unpopular girls are depressed, but over and above this there is no association of depressed mood and relational dissonance. Girls may be very adept at dealing with not being able to see eye-to-eye on a relationship compared with boys (for girls in such relations are the lowest on depressed mood), but girls are more affected by multiple nominations of dislike whereas for boys this is not problematic. We know from the significant popularity centralization effects for both dislike networks that there are indeed highly nominated disliked boys and girls, so it is not just a matter of different network structures explaining this difference. A possible explanation for this effect is that girls involved in relationally discrepant ties may be boundary spanners, already connected into their own groups but attempting to bridge across to other groups, the targets of which are likely to be non-depressed group leaders. In such a way, rejection of one party by the other extending a friendship is unlikely to be disastrous to mental health.

There are of course some limitations to the current research that suggest the need to be cautious about drawing conclusions from this study. We make no universal claims that these results hold in all contexts for adolescents. Our study involved one non-government-funded school and our findings may reflect a relatively homogenous, well-connected group of students with relatively good mental health. More heterogeneous groups of students in terms of social backgrounds, network ties and wellbeing may provide a different picture. Just as we had a relatively homogenous group in terms of socio-demographic backgrounds, a further limitation of the study is that we have not been able to explore the possible effects of cultural diversity and the students’ social networks and their wellbeing. Ethnic and racial identity has been shown to be related to race related stress and mental wellbeing (e.g. [66]). While it may be likely that our analysis masks differences between these groups, we could not assess this in this study. Our study included only six students who spoke a LOTE at home but of course this does not assess cultural diversity especially for second and third generation migrants. We did not ask the students what their backgrounds were and even if we had, it is likely we would have had insufficiently large groups of any one background to explore this question with any certainty. It would be interesting to further explore the likely effects on socio-demographic factors and ethnic backgrounds in a larger study involving more heterogeneous populations than in this one school.

Further, our sample contained young adolescents, and as the prevalence of depressed mood, particularly for girls, increases as they age, different results may arise from older adolescents. We have some missing data within the school, and we also have not included friends (and disliked peers) outside of school, though one school-based study which did not restrict nominations to *school friendships* reported only 5% of nominations were external to the school [67]. Finally, it is also important to remember that a young person’s social networks are not limited to peers; supportive relationships with parents and other adults are important and protective (e.g. [11]). Moving beyond cross-sectional data and into temporal analyses would lead to further insights into whether mental health outcomes result from social relations, or vice versa, or some combination of the two.

Nonetheless, the ability to simultaneously examine two social networks and to do so controlling for network self-organization moves beyond standard statistical approaches of examining social relationships, such as regression, and therefore is a considerable methodological advance. Our multivariate ERGM approach made considerable demands of our hypotheses, which had to compete with one another and other exogenous and endogenous network effects. The use of multivariate ERGM has permitted us to extend theory and test it by proposing a multi-network concept – relational dissonance – of how social relationships might be associated with mental health. This combination of negative and positive social network relations and their simultaneous measurement using statistical models for social networks is innovative, and we have demonstrated that using appropriate, albeit complex, methods for handling social network data provides useful insights into the relationships between social networks and young people’s wellbeing.

Our findings with respect to gender differences, in particular different responses to relational dissonance, indicates the complex nature of adolescents’ social relationships and the importance of appropriately assessing mental wellbeing not just the absence of depression/anxiety. Our study also has implications for the development of interventions aimed at promoting wellbeing and/or reducing depression. In particular, it has implications for how schools provide students with contexts and social and emotional skills for developing and maintaining social relationships, and structures and procedures for managing relational difficulties when they occur.
